# Modular Design of the Selectivity Filter Pore Loop in a Novel Family of Prokaryotic ‘Inward Rectifier’ (NirBac) channels

**DOI:** 10.1038/srep15305

**Published:** 2015-10-16

**Authors:** Lejla Zubcevic, Shizhen Wang, Vassiliy N. Bavro, Sun-Joo Lee, Colin G. Nichols, Stephen J. Tucker

**Affiliations:** 1 Clarendon Laboratory, Department of Physics, University of Oxford, Oxford, United Kingdom; 2 Washington University St. Louis, School Of Medicine, Centre for the Investigation of Membrane Excitability Diseases (CIMED), St. Louis, MO, USA; 3 OXION Initiative in Ion Channels and Disease, University of Oxford, Oxford, United Kingdom

## Abstract

Potassium channels exhibit a modular design with distinct structural and functional domains; in particular, a highly conserved pore-loop sequence that determines their ionic selectivity. We now report the functional characterisation of a novel group of functionally non-selective members of the prokaryotic ‘inward rectifier’ subfamily of K^+^ channels. These channels share all the key structural domains of eukaryotic and prokaryotic Kir/KirBac channels, but instead possess unique pore-loop selectivity filter sequences unrelated to any other known ionic selectivity filter. The strikingly unusual architecture of these ‘NirBac’ channels defines a new family of functionally non-selective ion channels, and also provides important insights into the modular design of ion channels, as well as the evolution of ionic selectivity within this superfamily of tetrameric cation channels.

Ion channels mediate rapid and selective movement of ions across the cell membrane, a process fundamental to nearly all forms of cellular electrical activity and cell signalling. One of the largest classes of ion channels is the superfamily of tetrameric cation channels, and within this superfamily, K^+^-selective channels are the most numerous^1^,^2^. Potassium channels encompass a number of functionally diverse subfamilies including voltage-gated K^+^ (Kv) channels, calcium-activated K^+^ (KCa) channels, as well as inwardly-rectifying (Kir) K^+^ channels. However, despite this functional diversity, all superfamily members share the same key signature sequence within the pore that creates a selective pathway for potassium ions across the membrane, often referred to as the pore-loop or ‘P-loop’^3^,^4^. The dichotomy between a highly conserved pore domain yet marked functional heterogeneity results from their modular structural design, in which varying evolutionary pressures appear to have fused the K^+^-selective transmembrane pore to a number of different regulatory domains to produce functionally distinct subfamilies (Fig. 1A)^2^,^5^,^6^.

The success of this modular architectural design within the tetrameric cation channel superfamily is highlighted by the fact that similar structures appear to have evolved different filters or P-loop segments that confer selectivity for other cations such as Na^+^ or Ca^2+^, or in some cases produce non-selective cation pores. There is some debate about the precise evolutionary relationship between P-loop segments in Na^+^ and Ca^2+^ channels^7^. However, evolution of the various K^+^ channel families appears much clearer through the fusion of different regulatory domains to an ‘ancestral’ 2-TM K^+^ selective filter module, exemplified by the KcsA and Kcv potassium channels^8^.

One example of such evolution is the family of inwardly-rectifying (Kir) potassium channels: the pore domain module remains highly conserved, but is fused to a cytoplasmic regulatory domain that confers the unique functional properties associated with these channels^9^,^10^. In eukaryotes, Kir channels are involved in maintenance of the resting membrane potential as well as a variety of K^+^-transport functions. Consequently, they are associated with the control of many vital physiological functions and their malfunction can result in a multitude of different diseases ranging from renal dysfunction, through various cardiac and neurological disorders, even to forms of diabetes^10^.

Genes encoding homologous (KirBac) channels are also found in many prokaryotic genomes^11^,^12^. The functional role of these prokaryotic KirBac channels remains less well-defined, but these homologs led to the first X-ray crystal structures of members of this family and have also enabled important insights into the structural features underlying Kir channel gating and regulation^13^,^14^,^15^,^16^,^17^. Eukaryotic Kir and prokaryotic KirBac channels share a remarkable degree of structural conservation throughout both their transmembrane regions and the K^+^-selective P-loop, as well as the large cytoplasmic ‘Kir’ domain, implying a relatively linear evolutionary path. We now report a group of novel prokaryotic KirBac channel homologs that possess P-loop segments with no clear parallels to any other known selectivity filters. This is highly unexpected and implies a previously unappreciated and constrained evolutionary route from an already evolved and functionally specialised K^+^ channel subunit to one with a completely novel pore that generates non-selective cation channels. These Kir domain-containing non-selective channels, which we therefore term ‘NirBacs’, provide a unique insight into the modular design and evolution of ionic selectivity within the superfamily of tetrameric cation channels.

## Results and Discussion

### Identification of NirBac channels

**Figure 1:**
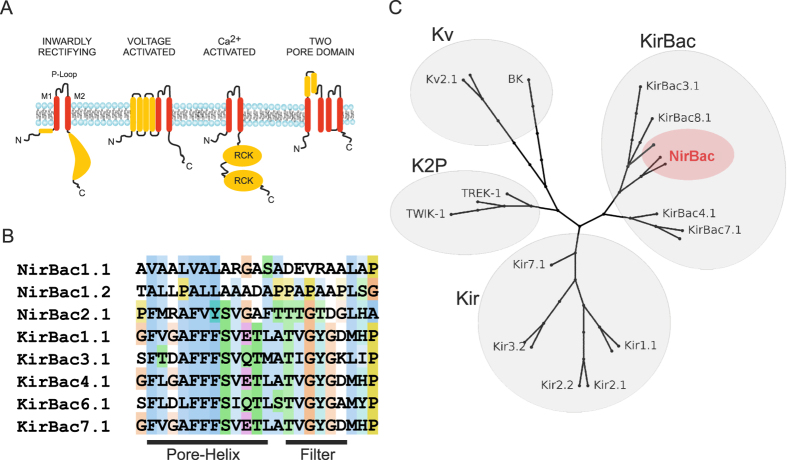
Evolution and modular design of K^+^ channel structures. (**A**) All K^+^ channels share a conserved P-loop region, which contains the selectivity filter and TM domains which provide passageway through the membrane. These conserved domains can be found fused to a number of different regulatory domains which results in a number of functionally different K^+^ channel families, including the inward rectifiers (Kir), voltage gated channels (Kv), calcium activated channels and Two-Pore channels (K2P). (**B**) An alignment of the P-loop regions of prokaryotic Kir channel homologues, KirBac, with the P-loop sequences of the novel inwardly rectifying channels, NirBac1.1, NirBac1.2 and NirBac2.1. (**C**) A phylogenetic tree showing that NirBac channels are placed firmly in the family of prokaryotic inward rectifiers.

Using KirBac1.1 as a query sequence in a BLAST search of the Integrated Microbial Genomes (IMG) Database we identified three novel genes with high homology throughout their overall sequence to the KirBac family of K^+^ channels, but which possess completely unique selectivity filter sequences (Fig. 1B,C and Supplementary Figure S1). Two of these homologs show marked variation throughout the entire P-loop sequence connecting TM2 and TM1, whilst in the third, the overall P-loop sequence appears conserved, although there is still marked divergence from the canonical K^+^ selectivity filter sequence (TxGYG). To verify these unique gene sequences, we obtained genomic DNA samples for each of the four organisms and PCR amplified each gene independently. Cloning and sequencing verified that the reported sequences within the database were 100% accurate and therefore confirmed the unique sequence of the predicted P-loop segments. The IMG database uses the Transporter Classification Database to assign these genes to the TC class A.1.2 inward rectifier K channel (IRK-C) family^18^, and due to their overall homology with other Kir/KirBac channels we consider this classification accurate. However, because of the non-canonical P-loop selectivity filter sequences, and their non-selective channel activities (see below), we refer to them as ‘NirBacs’ rather than KirBacs.

NirBac1.1 (YP_467386) was isolated from *Anaeromyxobacter dehalogenans* 2CP-C and shares 35% overall amino acid sequence identity (48% similarity) with KirBac1.1. However, there is no homology at all with KirBac1.1 throughout the entire P-loop segment. NirBac1.2 (YP_001381495) was found in the closely related strain *Anaeromyxobacter sp*. FW109-5 and shares 35% sequence identity (56% similarity) with KirBac1.1. Interestingly, the P-loop segment in NirBac1.2 shares no homology with the otherwise closely related NirBac1.1, despite >66% sequence identity throughout the rest of the protein. Two other closely-related sequences with P-loops identical to NirBac1.1 and >97% overall sequence identity were also identified in the strains *Anaeromyxobacter sp.*K (WP_012528270) and *Anaeromyxobacter dehalognenans* 2CP-1 (WP_015935350). Due to their almost complete identity to NirBac1.1, we did not examine them further. NirBac2.1 (YP_002762692) from *Gemmatimonas aurantiaca* T-27 shares approximately 35% overall identity with KirBac1.1. However, significant variation again occurs specifically within the functionally critical K^+^ selectivity filter sequence which reads TTGTD in NirBac2.1, and therefore deviates markedly from the consensus sequence found in all other K^+^-selective channels.

### Expression and Purification of NirBac Channels

The open reading frames of the three cDNAs were first codon optimised to reduce the very high (>75%) GC content found in their parental genomes, and then expressed in *E. coli*. Expression and purification of NirBac2.1 proved problematic and was not pursued further. However, both NirBac1.1 and NirBac1.2 expressed protein at levels sufficient for purification and characterisation (Supplementary Figure S2A). Previous studies have shown that the consensus TxGYG K^+^-selectivity filter sequence within the P-loop plays a significant role in stabilising the tetrameric assembly in a K^+^ channel. Tetramer stability has also been shown to be controlled by ion binding within the selectivity filter of both Kv and Kir channels^19^,^20^. We therefore analysed the oligomeric state of these NirBac channels using size-exclusion chromatography and gel electrophoresis. It was found that both NirBac1.1 and NirBac1.2 behaved as monomers in denaturing environments, but consistent with a tetrameric assembly both formed stable oligomers of ~120 kDa, under non-denaturing conditions (Supplementary Figure S2B,C). These results indicate that NirBac channels are able to assemble into stable tetramers even in the absence of a TxGYG selectivity filter and may therefore form functional channels.

### Lipid dependence of functional expression

**Figure 2:**
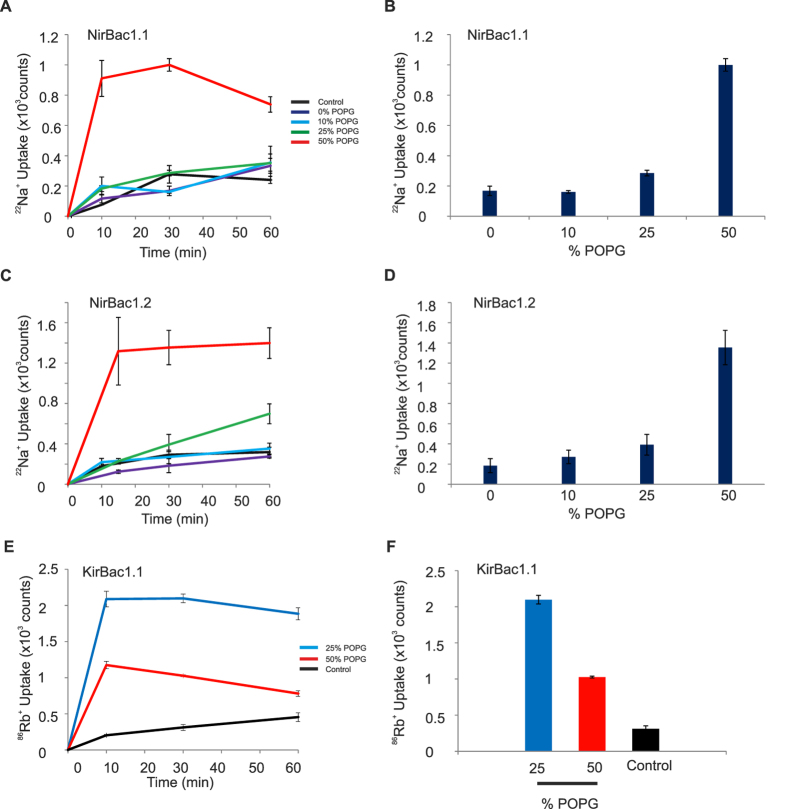
NirBac channel activity is lipid-dependent. (**A**) Time-course of ^22^Na^+^ uptake mediated by NirBac1.1 driven by Na^+^. The purified protein was reconstituted into liposomes containing 0–50% POPG and the uptake monitored over 60 minutes. The activity increases significantly only when the POPG content is raised to 50%. (**B**) ^22^Na^+^ uptake shown at steady-state (30 minutes). Error bars represent s.e.m., *n* = 3. (**C**) Time-course of ^22^Na^+^ uptake for NirBac1.2. The activity of the channel is also significantly increased in liposomes containing 50% POPG. (**D**) ^22^Na^+^ uptake at steady-state measured in liposomes containing 0–50% POPG. Error bars represent s.e.m., and *n* = 6–8. (**E**) Time course for ^86^Rb^+^ uptake mediated by KirBac1.1 in the presence of 25% POPG and 50% POPG. Error bars represent s.e.m. and *n* = 3 for all timepoints. (**F**) Steady state ^86^Rb^+^ uptake (t = 30 minutes).

To examine the functional properties of these unique channel proteins, we reconstituted purified proteins into liposomes and carried out flux assays similar to those described previously^15^,^21^,^22^,^23^. Due to the lack of a consensus K^+^ selective filter sequence we used ^22^Na^+^ as the tracer ion in these assays. Initially we found that only low levels of ^22^Na^+^ uptake could be measured using either NirBac1.1 or NirBac1.2. However, we observed that flux activity was highly dependent upon the relative POPG content of the liposomes and flux increased more than 5-fold when the POPG content was raised from 25% to 50% of total lipid content (Fig. 2A–D). To test whether this sensitivity to anionic phospholipids was unique to NirBac channels, we also examined the activity of KirBac1.1 channel in liposomes containing either 25% or 50% POPG. Interestingly, in direct contrast to NirBac1.1 and NirBac1.2, the functional activity of KirBac1.1 was significantly inhibited by high POPG concentrations (Fig. 2E,F).

### Ionic Selectivity

**Figure 3:**
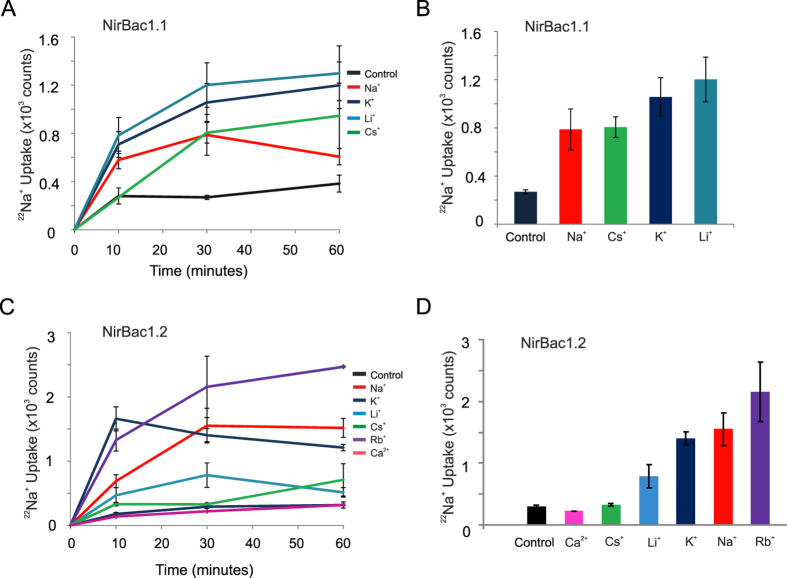
Ionic selectivity in NirBac channels. This was assessed by the ability of cations to drive the uptake of ^22^Na^+^. (**A**,**B**) Time-course and steady-state uptake of ^22^Na^+^ driven by Na^+^, K^+^, Li^+^ and Cs^+^ ions. In NirBac1.1 all ions drive the uptake of the radioactive tracer to a similar extent, indicating that the channel is non-selective. (**C**,**D**) The same experiment performed to determine the selectivity of NirBac1.2. NirBac1.2 appears to favour permeation of Na^+^, K^+^ and Rb^+^ over Cs^+^ and Li^+^ (*p* < 0.05), and it is not permeant to Ca^2+^. Error bars represent s.e.m., *n* = 3–21.

We next reconstituted NirBac1.1 and NirBac1.2 proteins into 1:1 POPE:POPG liposomes and measured the relative uptake of ^22^Na^+^ driven by a range of different cations incorporated within the liposomes. For NirBac1.1 we found that Na^+^, K^+^, Rb^+^, Cs^+^ and Li^+^ gradients all supported similar levels of ^22^Na^+^ uptake suggesting that this channel exhibits little ionic selectivity (Fig. 3A,B). NirBac1.2 appeared to exhibit a small preference for Na^+^, K^+^ and Rb^+^ ions over Cs^+^ and Li^+^, but cation permeability appears limited to monovalent cations as NirBac1.2 was not permeable to Ca^2+^ (Fig. 3C,D).

To further probe the interaction of the NirBac1.2 pore with monovalent cations, we utilised an additional approach to assess selectivity. In the liposomal flux assays used above, the principal driving force for ^22^Na^+^ uptake is the large electrical gradient created by the efflux of permeant cations through the reconstituted channel. Addition of cations to the extraliposomal solution will dissipate this gradient and decrease ^22^Na^+^ uptake in proportion to the permeability of the added ion. This therefore provides an alternative measure of relative permeability, and consistent with our previous experiments, we found that addition of 1 mM Na^+^, K^+^ or Rb^+^ to the extraliposomal buffer resulted in significant reduction in ^22^Na^+^ uptake. Li^+^ also markedly inhibited ^22^Na^+^ uptake (Supplementary Figure S3) although this particular ion exhibited little permeability in the previous assays, which suggests that Li^+^ may interact directly with the NirBac1.2 pore to produce a permeant block. Cs^+^ ions, which also failed to permeate in the previous assay produced only weak inhibition of ^22^Na^+^ uptake.

KirBac1.1 and KirBac3.1 channel activity has previously been shown to be inhibited by acidic pH^15^,^17^. We therefore examined the effect of changing pH on NirBac channel activity and found that ^22^Na^+^ uptake was reduced by an increase in [H^+^] (Supplementary Figure S4A). We next examined the effect of several known Kir channel blockers such as polyamines (spermine), quaternary ammonium ions and Ba^2+^ (Supplementary Figure S4B–D). These blockers work asymmetrically in eukaryotic channels^10^, and similar to previous studies of prokaryotic channels^19^,^20^,^21^,^22^,^23^, we assume the NirBac channels orient randomly in the liposomes and so non-permeant blockers were added to both sides of the membrane. However, we found that none of these blockers had any significant effect on ^22^Na^+^ uptake.

### Properties of the NirBac1.1 TM/Pore domain

**Figure 4:**
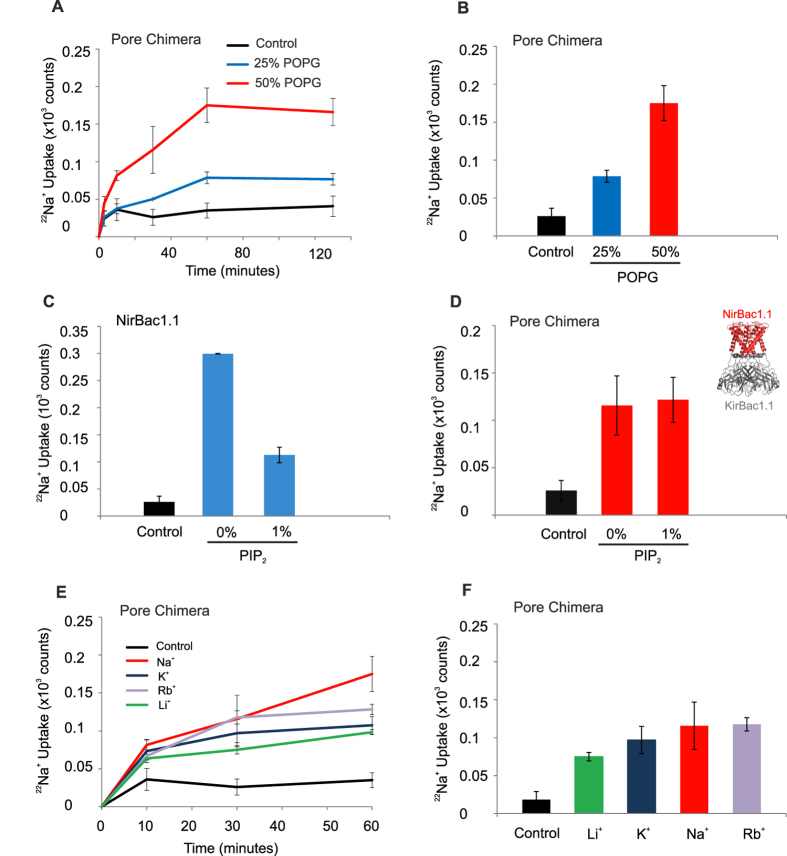
Role of NirBac1.1 pore. A chimera was engineered to contain the TM domains of NirBac1.1 and the CTD domains of KirBac1.1. (**A**,**B**) The pore chimera is sensitive to liposomal POPG concentrations. The time-course and steady-state measurements of ^22^Na^+^ uptake (driven by Na^+^) show a significant increase in activity when the POPG concentration is raised to 50%. (**C**) NirBac1.1 is inhibited by 1% PIP_2_ in 1:1 POPE:POPG liposomes. Bars represent ^22^Na^+^ uptake at 30 minutes (steady-state). (**D**) The pore chimera appears insensitive to inhibition by 1% PIP_2_. Inset shows regions exchanged in this chimera; see supplementary Fig S4 for details. (**E**,**F**) Consistent with the properties of the NirBac1.1 pore, the chimera is permeant to both Na^+^, K^+^, Rb^+^ and Li^+^ to a similar extent. All error bars represent s.e.m., *n* = 3.

POPG is a necessary co-factor in the PIP_2_ dependent activation of eukaryotic Kir2.x channels where a distinct POPG binding site is located on the cytoplasmic side of the TMD/CTD interface^24^. In order to determine whether the profound effects of POPG on NirBac channel activity may be structurally equivalent to the effects of POPG in eukaryotic Kir channels, we examined the functional properties of a chimera consisting of the NirBac1.1 TM/Pore domain and the cytoplasmic domains of the archetypal KirBac channel, KirBac1.1. This chimeric channel was successfully expressed and purified (Supplementary Figure S5) and its activity tested in flux assays. Like the parental NirBac channel, the functional activity of this chimera increased with the relative proportion of POPG in the liposomes (Fig. 4A,B) indicating that the structural elements responsible for up-regulation by this negatively charged phospholipid are located within the modular TM/Pore domains of NirBac1.1.

The activity of Kir and KirBac channels is also strongly regulated by another anionic phospholipid, phosphatidylinositol-4,5-bisphosphate (PIP_2_), but unlike eukaryotic Kir channels, KirBac1.1 activity is inhibited by PIP_2_ 25,26,27. Wild-type NirBac1.1 channel was also inhibited by 1% PIP_2_ (Fig. 4C). However, the NirBac/KirBac chimera was insensitive to PIP_2_ inhibition (Fig. 4D), despite the fact that both parental channels (KirBac1.1 and NirBac1.1) are strongly inhibited by PIP_2_. The chimeric junctions between NirBac1.1 and KirBac1.1 are located at the interface between the transmembrane and cytoplasmic domains i.e. within the regions likely to interact with PIP_2_. It is therefore possible that the PIP_2_ inhibitory site is compromised in the chimera. Nevertheless, the chimera retains activation by POPG indicating that the effects of these two anionic phospholipids remain structurally and functionally distinct^24^.

Finally, we examined the ionic selectivity of the chimera. Consistent with the properties of the parental NirBac1.1 pore, the chimera appeared to be non-selective with Li^+^, Na^+^, K^+^ and Rb^+^ permeating equally well (Fig. 4E,F).

### Evolutionary Implications

Our identification and functional characterisation of these unique non-selective NirBac channels demonstrates a previously unrecognised type of modular evolution within the K^+^ channel family in which the overall Kir/KirBac architecture remains highly conserved, but in which there are major changes within the P-loop filter segment. Many previous studies of tetrameric cation channels have highlighted the modular construction^4^,^6^, but within this superfamily, modular changes of the entire P-loop segment appear to occur primarily within members of the 6-TM voltage-gated channel branch^7^. The NirBac channels therefore represent one of the first examples in which functionally critical changes in the P-loop segment have been observed within another major branch of this superfamily.

The evolution of functionally different P-loop segments within the conserved architectural framework of the 6-TM voltage-gated branch of the superfamily is well documented, and encompasses channels that can be selective for Na^+^, Ca^2+^ or even non-selective between Na^+^/K^+ ^28. However, the structural and functional homology between such members of the 6-TM branch of the superfamily is significantly more diverse than between NirBac and KirBac channels which differ only within the P-loop region. The modularity of selectivity filter design in this superfamily is also evident in the CNG and HCN channels. The prokaryotic CNG channel MloK possesses a canonical K^+^-selective P-loop, but its eukaryotic counterparts are non-selective due to subtle modifications within the canonical TxGYG filter^29^,^30^. These changes are similar to those found in the filter of the 2-TM non-selective prokaryotic NaK channel^28^,^31^. However, in all of these other non-selective channels, the P-loop segments still share a degree of overall homology with the canonical K^+^-selective P-loop segments and the other parts of their structures are also as diversified as the P-loop segments.

This suggests that there must have been major evolutionary pressure applied for the P-loops within these NirBac channels to have evolved so strikingly from the classical K^+^ channel structures, possibly reflecting the specialised environments of these bacteria. This modularity of the P-loop segment in the NirBac channels is most evident when comparing the NirBac1.1 and NirBac1.2 sequences which share no sequence homology at all within their P-loop segments, even though they possess >66% sequence identity throughout the rest of their structure. Furthermore, the P-loop segments of these NirBac channels share no obvious homology with any known selectivity filter sequence, and so their origin remains unknown.

It also remains unclear why different strains of *Anaeromyxobacter* should have exploited this modular design structure to produce two otherwise almost identical channels (NirBac1.1 and NirBac1.2) but with such unique selectivity filters. Interestingly, current annotation of the *Anaeromyxobacter* genomes does not reveal any other obvious K^+^ channel genes, although they do contain a range of different cation-selective transport genes. It is therefore possible that the P-loop segments of these NirBac channels may represent an evolutionary hotspot. It has been reported that alternative splicing within the P-loop of the invertebrate NACLN cation channel regulates selectivity^32^, but this is the first report of such specific modular changes within a prokaryotic cation channel.

As is the case for the majority of other prokaryotic ion channels, the *in vivo* functional role of bacterial Kir domain-containing channels remains unclear^33^. A gene-knockout study of KirBac6.1 in the cyanobacterium *Synechocystis* revealed a modest K^+^-dependent growth phenotype^23^, but in many cases the role of microbial channels may only become apparent under conditions of extreme stress. *Anaeromyxobacter* is a δ-proteobacterium isolated from uranium contaminated soils, and there is considerable interest in these bacteria due to their potential role in bioremediation^34^,^35^,^36^. Considering the exotic environments that these anaerobic bacteria inhabit, as well as their sophisticated chemistry, it is therefore possible that the P-loop segments in these NirBac channels may have evolved to adapt to new environmental demands.

### Unique features of the NirBac channels

The selectivity filters of NirBac1.1 and NirBac1.2 are both non-selective, yet there are marked functional differences between them. The pore of NirBac1.1 appears permeant to Na^+^, K^+^, Li^+^ and Cs^+^ ions. By contrast, NirBac1.2 appears to be more selective for Na^+^, K^+^ and Rb^+^, than Cs^+^, whilst Li^+^ appears to act as a permeant blocker. Activation of the NirBac channels by the anionic phospholipid POPG is also intriguing; Our finding that both NirBac and the NirBac/KirBac chimera are activated by POPG suggests the presence of a lipid regulation site in the TM region of the NirBac channel that is distinct from the POPG binding site identified in eukaryotic Kir2.x channels^24^.

In summary, we have identified a novel family of non-selective ‘inward-rectifier’ NirBac channels. The structural and functional features of these channels offer a unique perspective on the evolution of the P-loop in the superfamily of tetrameric cation channels. Our demonstration of such a highly modular evolution of the P-loop segment provides a template that might be exploited in future efforts to engineer a switched, or even completely novel, ion selectivity in other members of this superfamily of cation channels, for therapeutic control of cellular excitability. Also, further structural studies of these channels, and the unique P-loops they contain, may provide insight into the molecular mechanisms that control selective permeation through different classes of ion channel.

## Methods

### Molecular Biology

KirBac1.1 was used a query protein sequence to search the Integrated Microbial Genomes Database (https://img.jgi.doe.gov). After identification, NirBac1.1 (YP_467386) was isolated from a genomic DNA sample of *Anaeromyxobacter dehalogenans* 2CP-C, NirBac1.2 (YP_001381495) from *Anaeromyxobacter sp. Fw109-5*, and NirBac2.1 (YP_002762692) from *Gemmatimonas aurantiaca* T-27. Genomic DNA samples for all three organisms were obtained through the IMG Database. Independent sequencing of all three genes confirmed the accuracy of the sequences identified in the database, but due to their high GC content they were not well-suited for expression in *E. coli* and codon-optimised versions of all three NirBac genes were therefore synthesised (GenScript USA Inc.).

### Expression and Purification

Codon optimised NirBac channel genes in the pQE60Lac vector (NirBac1.2 and KirBac1.1) or pET28a vector (NirBac1.1) were expressed in Rosetta pLysS *E. coli* (Novagen). Cell pellets were resuspended in 25 ml per 5 g bacterial pellet in a buffer of 50 mM Tris pH 7.8,150 mM NaCl (150 mM LiCl), complemented with one tablet of EDTA free protease cocktail (Roche), 2 g/ml DNAseI (Sigma Aldrich) and 0.5 mg/ml lysozyme (Fluka). The resuspension was passed through a cell disruptor 4–6 times (Stansted Fluid Power Ltd) at a pressure of ~12000 psi. The lysate was centrifuged for 10 minutes at 7,000 RCF to remove large debris and unbroken cells. The supernatant was transferred into ultracentrifuge tubes, and spun at 200,000 RCF for 90 minutes. Membrane pellets were resuspended and washed in 50 mM Tris pH 7.8, 1 M NaCl (1 M LiCl) before being centrifuged for another hour at 200,000 RCF. An additional wash cycle was performed in 50 mM Tris pH 7.8, 150 mM NaCl (150 mM LiCl). The washed membranes were weighed and resuspended in 20 ml per gram membranes 50 mM Tris pH 7.8, 150 mM NaCl (150 mM LiCl), 30 mM Dodecyl-β-Maltoside (DDM, Affymetrix). The membranes were resuspended in detergent buffer and incubated for 3 hours on a rotating wheel at 4 °C. Following solubilisation, the membranes were centrifuged at 70,000 RCF for 30 minutes to remove debris and unsolubilised material. To the cleared solubilised membranes 10 mM imidazole was added before they were loaded on cobalt TALON affinity resin (Takara). The binding was performed overnight on a rotating wheel at 4 °C. The unbound material was removed by gravity flow using a glass column. The resin was washed with 25 times column volume (cv) of each of the following wash buffers: Wash 1. 50 mM Tris pH 7.8, 150 mM NaCl (150 mM LiCl), 10 mM imidazole, 2 mM DDM (1 mM TriDM); Wash 2. 50 mM Tris pH 7.8, 500 mM NaCl (500 mM LiCl), 20 mM imidazole, 2 mM DDM (1 mM TriDM); Wash 3. Tris pH 7.8, 150 mM NaCl (150 mM LiCl), 80 mM imidazole, 2 mM DDM (1 mM TriDM). The channel was eluted from the TALON resin in 2 cv elution buffer consisting of: 50 mM Tris pH 7.8, 150 mM NaCl (150 mM LiCl), 400 mM imidazole, 1 mM DDM (0.5 mM TriDM). The purified channel protein was run on a size exclusion column (Superdex200, GE Healthcare) and the tetrameric fraction collected.

### Liposomal flux assays

POPE and POPG lipids solubilized in CHAPS detergent were mixed in a 1:1 ratio to a final concentration of 1 mg/ml. 10 μg protein was added to 95 μl lipid mixture and incubated at room temperature for 30 minutes. Sephadex G50 columns equilibrated with buffer B (10 mM HEPES (substituted with 10 mM MES (2-(N-morpholino)ethanesulfonic acid) or 10 mM CAPSO (N-cyclohexyl-3-aminopropanesulfonic acid) in experiments that analysed the pH-dependence of the channel) 450 mM NaCl (substituted with KCl, RbCl, LiCl, CsCl, or CaCl_2_, where appropriate), 4 mM NMDG. Buffer pH was adjusted to the stated pH with NMDG. Columns were partially dehydrated by centrifugation for 10 seconds at 1000 RCF. The mixture of lipids and protein was loaded on the column and then centrifuged at 700 RCF for 10 seconds to remove CHAPS detergent. The flow-through containing the newly formed proteoliposomes was collected. To remove external ions the liposomes were filtered through a partially dehydrated Sephadex column equilibrated in buffer C (10 mM HEPES, 400 mM sorbitol, 4 mM NMDG). To each proteoliposome sample 420 μl buffer C complemented with ^22^Na^+^ was added and 60 μl samples were taken out at the stated time points. To remove external ^22^Na^+^, samples were run through a Dowex column. The radioactive content of the proteoliposomes (counts per minute; c.p.m.) was measured in a scintillation counter. For the studies of quaternary ammonium ion block, either Tetrapentylammonium (TPA) or Tetrahexylammonium (THA) were added to both Buffer B and Buffer C (i.e both inside and outside). For barium/spermine block, the indicated concentrations of barium chloride were added to Buffer C, and spermine to both Buffer B and C.

### Alignments and Phylogenetic Analysis

Alignments were made using the Expresso T-Coffee system, where sequences are aligned with the help of PDB entries^37^. The resulting alignment was used to construct a maximum likelihood unrooted phylogenetic tree. Alignment figures were prepared using Jalview^38^.

## Additional Information

**How to cite this article**: Zubcevic, L. *et al*. Modular Design of the Selectivity Filter Pore Loop in a Novel Family of Prokaryotic 'Inward Rectifier' (NirBac) channels. *Sci. Rep.* **5**, 15305; doi: 10.1038/srep15305 (2015).

## Supplementary Material

Supplementary Information

## References

[b1] Hille B. In Ion Channels of Excitable Membranes , 3^rd^ edn. Ch 3, 72–73 (Sinauer Associates, 2001).

[b2] Yu F. H. & Catterall W. A. The VGL-chanome: a protein superfamily specialized for electrical signaling and ionic homeostasis. Sci STKE 2004, re15 (2004). 15467096 10.1126/stke.2532004re15

[b3] MacKinnon R. Pore loops: an emerging theme in ion channel structure. Neuron 14, 889–892 (1995). 7538310 10.1016/0896-6273(95)90327-5

[b4] Heginbotham L., Abramson T. & MacKinnon R. A functional connection between the pores of distantly related ion channels as revealed by mutant K+ channels. Science 258, 1152–1155 (1992). 1279807 10.1126/science.1279807

[b5] Choe S. Potassium channel structures. Nat Rev Neurosci 3, 115–121 (2002). 11836519 10.1038/nrn727

[b6] Lu Z., Klem A. M. & Ramu Y. Ion conduction pore is conserved among potassium channels. Nature 413, 809–813 (2001). 11677598 10.1038/35101535

[b7] Charalambous K. & Wallace B. A. NaChBac: the long lost sodium channel ancestor. Biochemistry 50, 6742–6752 (2011). 21770445 10.1021/bi200942y PMC3153336

[b8] Thiel G., Moroni A., Blanc G. & Van Etten J. L. Potassium ion channels: could they have evolved from viruses? Plant Physiol 162, 1215–1224 (2013). 23719891 10.1104/pp.113.219360 PMC3707557

[b9] Bichet D., Haass F. A. & Jan L. Y. Merging functional studies with structures of inward-rectifier K(+) channels. Nat Rev Neurosci 4, 957–967 (2003). 14618155 10.1038/nrn1244

[b10] Hibino H. . Inwardly rectifying potassium channels: their structure, function, and physiological roles. Physiol Rev 90, 291–366 (2010). 20086079 10.1152/physrev.00021.2009

[b11] Durell S. R. & Guy H. R. A family of putative Kir potassium channels in prokaryotes. BMC Evol Biol 1, 14 (2001). 11806753 10.1186/1471-2148-1-14 PMC64639

[b12] Sun S., Gan J. H., Paynter J. J. & Tucker S. J. Cloning and functional characterization of a superfamily of microbial inwardly rectifying potassium channels. Physiol Genomics 26, 1–7 (2006). 16595742 10.1152/physiolgenomics.00026.2006

[b13] Bavro V. N. . Structure of a KirBac potassium channel with an open bundle crossing indicates a mechanism of channel gating. Nat Struct Mol Biol 19, 158–163 (2012). 22231399 10.1038/nsmb.2208 PMC3272479

[b14] Clarke O. B. . Domain reorientation and rotation of an intracellular assembly regulate conduction in Kir potassium channels. Cell 141, 1018–1029 (2010). 20564790 10.1016/j.cell.2010.05.003

[b15] Enkvetchakul D. . Functional characterization of a prokaryotic Kir channel. J Biol Chem 279, 47076–47080 (2004). 15448150 10.1074/jbc.C400417200 PMC8629170

[b16] Kuo A. . Crystal structure of the potassium channel KirBac1.1 in the closed state. Science 300, 1922–1926 (2003). 12738871 10.1126/science.1085028

[b17] Zubcevic L. . Control of KirBac3.1 potassium channel gating at the interface between cytoplasmic domains. J Biol Chem 289, 143–151 (2014). 24257749 10.1074/jbc.M113.501833 PMC3879539

[b18] Saier M. H. Jr., Reddy V. S., Tamang D. G. & Vastermark A. The transporter classification database. Nucleic Acids Res 42, D251–258 (2014). 24225317 10.1093/nar/gkt1097 PMC3964967

[b19] Krishnan M. N. . Functional role and affinity of inorganic cations in stabilizing the tetrameric structure of the KcsA K+ channel. J Gen Physiol 126, 271–283 (2005). 16129774 10.1085/jgp.200509323 PMC2266582

[b20] Wang S. . Differential roles of blocking ions in KirBac1.1 tetramer stability. J Biol Chem 284, 2854–2860 (2009). 19033439 10.1074/jbc.M807474200 PMC2631979

[b21] Cheng W. W., Enkvetchakul D. & Nichols C. G. KirBac1.1: it’s an inward rectifying potassium channel. J Gen Physiol 133, 295–305 (2009). 19204189 10.1085/jgp.200810125 PMC2654083

[b22] McCusker E. C. . Structure of a bacterial voltage-gated sodium channel pore reveals mechanisms of opening and closing. Nat Commun 3, 1102 (2012). 23033078 10.1038/ncomms2077 PMC3493636

[b23] Paynter J. J. . Functional complementation and genetic deletion studies of KirBac channels: activatory mutations highlight gating-sensitive domains. J Biol Chem 285, 40754–40761 (2010). 20876570 10.1074/jbc.M110.175687 PMC3003375

[b24] Lee S. J. . Secondary anionic phospholipid binding site and gating mechanism in Kir2.1 inward rectifier channels. Nat Commun 4, 2786 (2013). 24270915 10.1038/ncomms3786 PMC3868208

[b25] D’Avanzo N., Cheng W. W., Doyle D. A. & Nichols C. G. Direct and specific activation of human inward rectifier K+ channels by membrane phosphatidylinositol 4,5-bisphosphate. J Biol Chem 285, 37129–37132 (2010). 20921230 10.1074/jbc.C110.186692 PMC2988318

[b26] Enkvetchakul D., Jeliazkova I. & Nichols C. G. Direct modulation of Kir channel gating by membrane phosphatidylinositol 4,5-bisphosphate. J Biol Chem 280, 35785–35788 (2005). 16144841 10.1074/jbc.C500355200

[b27] D’Avanzo N. . Lipids driving protein structure? Evolutionary adaptations in Kir channels. Channels 4, 139–141 (2010). 21150302 10.4161/chan.4.3.12129 PMC3241986

[b28] Alam A. & Jiang Y. Structural studies of ion selectivity in tetrameric cation channels. J Gen Physiol 137, 397–403 (2011). 21518828 10.1085/jgp.201010546 PMC3082925

[b29] Craven K. B. & Zagotta W. N. CNG and HCN channels: two peas, one pod. Annu Rev Physiol 68, 375–401 (2006). 16460277 10.1146/annurev.physiol.68.040104.134728

[b30] Nimigean C. M., Shane T. & Miller C. A cyclic nucleotide modulated prokaryotic K+ channel. J Gen Physiol 124, 203–210 (2004). 15337819 10.1085/jgp.200409133 PMC2233883

[b31] Shi N. . Atomic structure of a Na+- and K+-conducting channel. Nature 440, 570–574 (2006). 16467789 10.1038/nature04508

[b32] Senatore A. . NALCN ion channels have alternative selectivity filters resembling calcium channels or sodium channels. PLoS One 8, e55088 (2013). 23383067 10.1371/journal.pone.0055088 PMC3557258

[b33] Kuo M. M. . Prokaryotic K(+) channels: from crystal structures to diversity. FEMS Microbiol Rev 29, 961–985 (2005). 16026885 10.1016/j.femsre.2005.03.003

[b34] Cardenas E. . Microbial communities in contaminated sediments, associated with bioremediation of uranium to submicromolar levels. Appl Environ Microbiol 74, 3718–3729 (2008). 18456853 10.1128/AEM.02308-07 PMC2446554

[b35] Marshall M. J. . Electron donor-dependent radionuclide reduction and nanoparticle formation by Anaeromyxobacter dehalogenans strain 2CP-C. Environ Microbiol 11, 534–543 (2009). 19196283 10.1111/j.1462-2920.2008.01795.x

[b36] Thomas S. H. . Diversity and distribution of anaeromyxobacter strains in a uranium-contaminated subsurface environment with a nonuniform groundwater flow. Appl Environ Microbiol 75, 3679–3687 (2009). 19346346 10.1128/AEM.02473-08 PMC2687297

[b37] Taly J. F. . Using the T-Coffee package to build multiple sequence alignments of protein, RNA, DNA sequences and 3D structures. Nat Protoc 6, 1669–1682 (2011). 21979275 10.1038/nprot.2011.393

[b38] Waterhouse A. M. . Jalview Version 2–a multiple sequence alignment editor and analysis workbench. Bioinformatics 25, 1189–1191 (2009). 19151095 10.1093/bioinformatics/btp033 PMC2672624

