# Supplementary material for: Modular Design of the Selectivity Filter Pore Loop in a Novel Family of Prokaryotic ‘Inward Rectifier’ (NirBac) channels
Source: Sci Rep. 2015 Oct 16;5:15305. doi: 10.1038/srep15305 (PMC4607889; doi:10.1038/srep15305)
Supplement: Supplementary Information [file srep15305-s1.pdf]

SUPPLEMENTARY INFORMATION

Modular Design of the Selectivity Filter Pore Loop in a Novel Family of Prokaryotic 'Inward Rectifier' (NirBac) channels

Lejla Zubcevic, Shizhen Wang, Vassiliy N. Bavro, Sun-Joo Lee, Colin G. Nichols

and Stephen J. Tucker

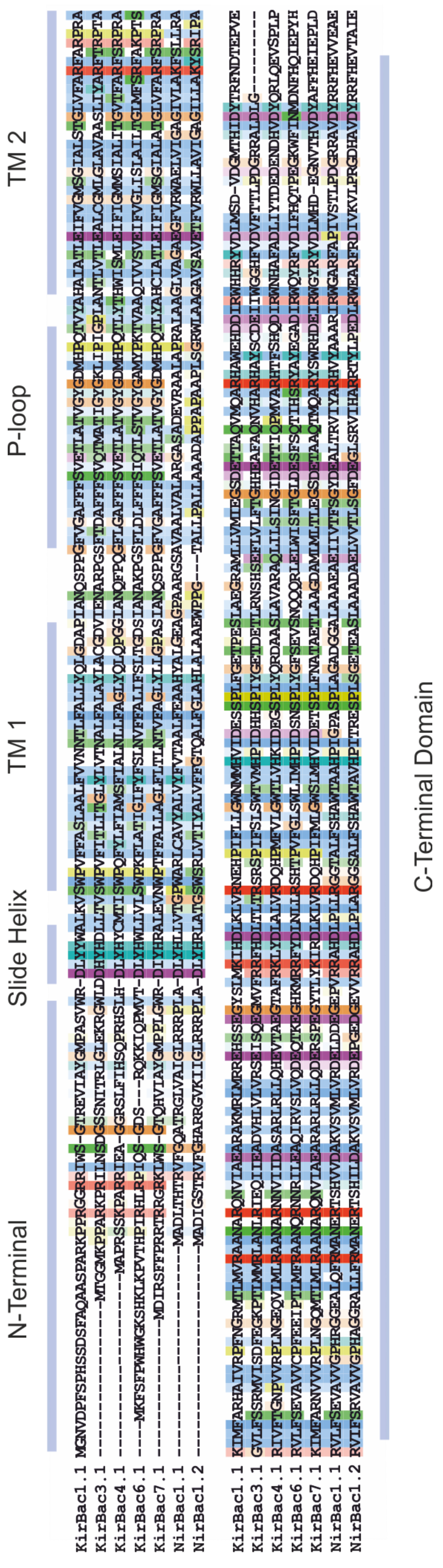

Figure S1 Full sequence alignment of KirBac and NirBac channels.

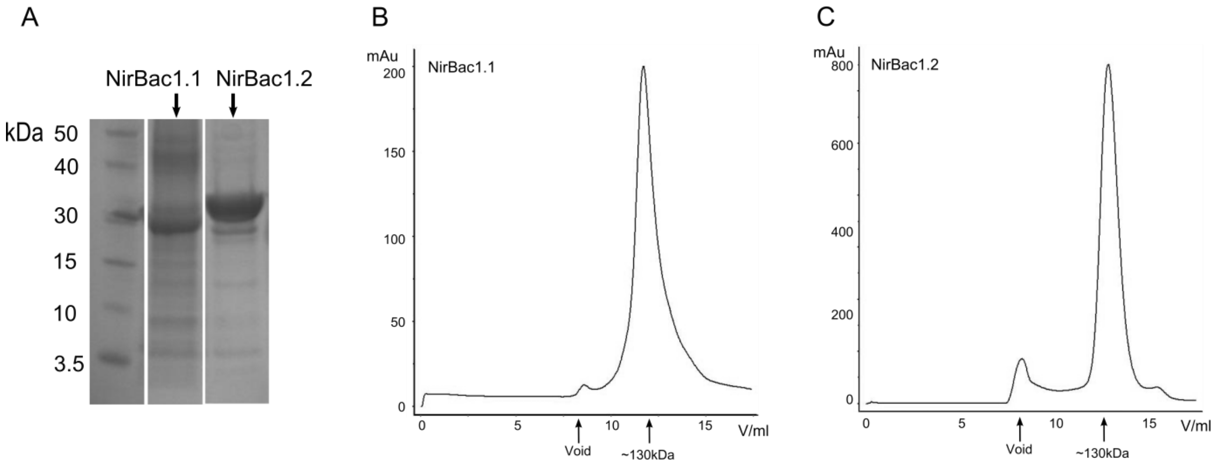

Figure S2 The oligomeric state of NirBac. **A** The purified proteins were run on an SDS-PAGE gel. Under denaturing conditions both proteins migrate at ~30kDa, which is the predicted M_w for a monomer. **B** and **C** Size exclusion chromatography profiles for the NirBac channels suggest that under non-denaturing conditions the proteins form stable tetrameric assemblies.

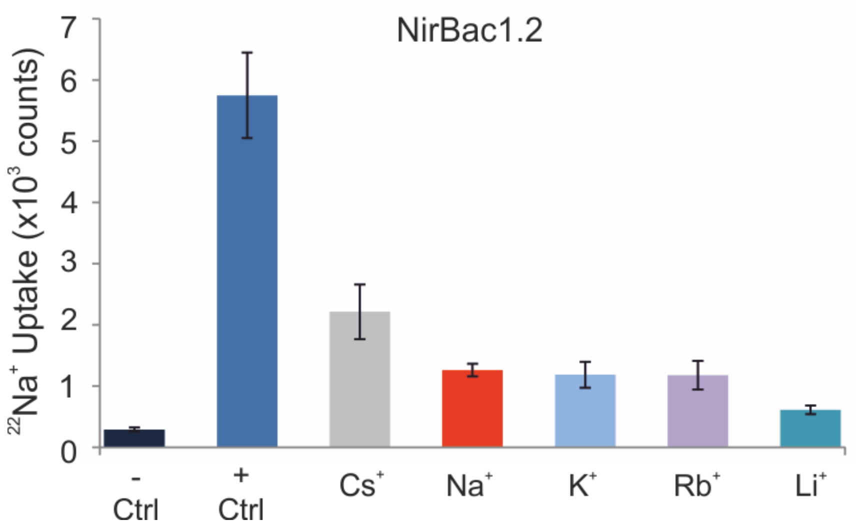

Figure S3 NirBac1.2 selectivity was further tested by addition of 1mM Cs^+, Na^+, K^+, Rb^+, and Li^+ to the extraliposomal buffer. The effect on the sodium-driven flux reflects the relative selectivity of the channel for sodium over the tested cations. Consistent with the previous experiment, Na^+, K^+ and Rb^+ all inhibit sodium driven flux to a similar extent. By contrast, Li^+, which permeates poorly, appears to be the most potent inhibitor which suggests a strong interaction with the NirBac1.2 pore. Error bars represent +/- SEM, and n=3.

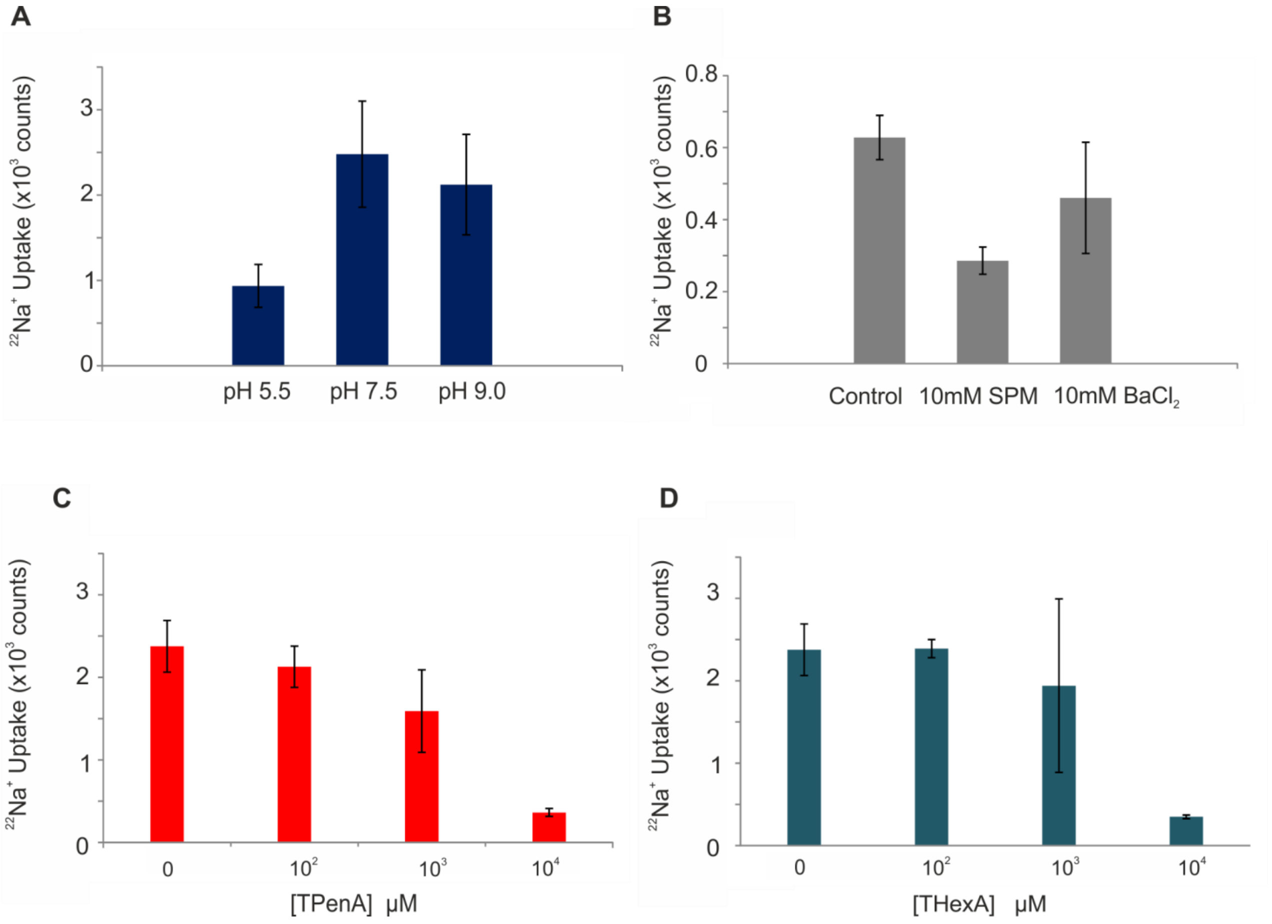

Figure S4 A The effect of pH on activity of NirBac1.2. In comparison to pH 7.5, channel activity was decreased at acidic pH 5.5, but no effect of alkalinisation was observed. pH was changed in both the intra- and extra-liposomal buffers. **B–D** The effect of known Kir channel blockers on NirBac1.2. Spermine (10 mM) had a modest effect on channel activity, but Ba^{2+} ions (10 mM) did not significantly interfere with $^{22}Na^{+}$ uptake. Spermine and barium were applied to the outside of the liposomes. Neither of the quaternary ammonium ion blockers, Tetrapentylammonium (TPA) or Tetrahexylammonium (THA) inhibited $^{22}Na^{+}$ uptake. TPA and THA were applied both to the inside and outside of the liposomes. Inhibition was only observed at very high concentrations (>1 mM). Error bars represent +/- SEM, and n=3.

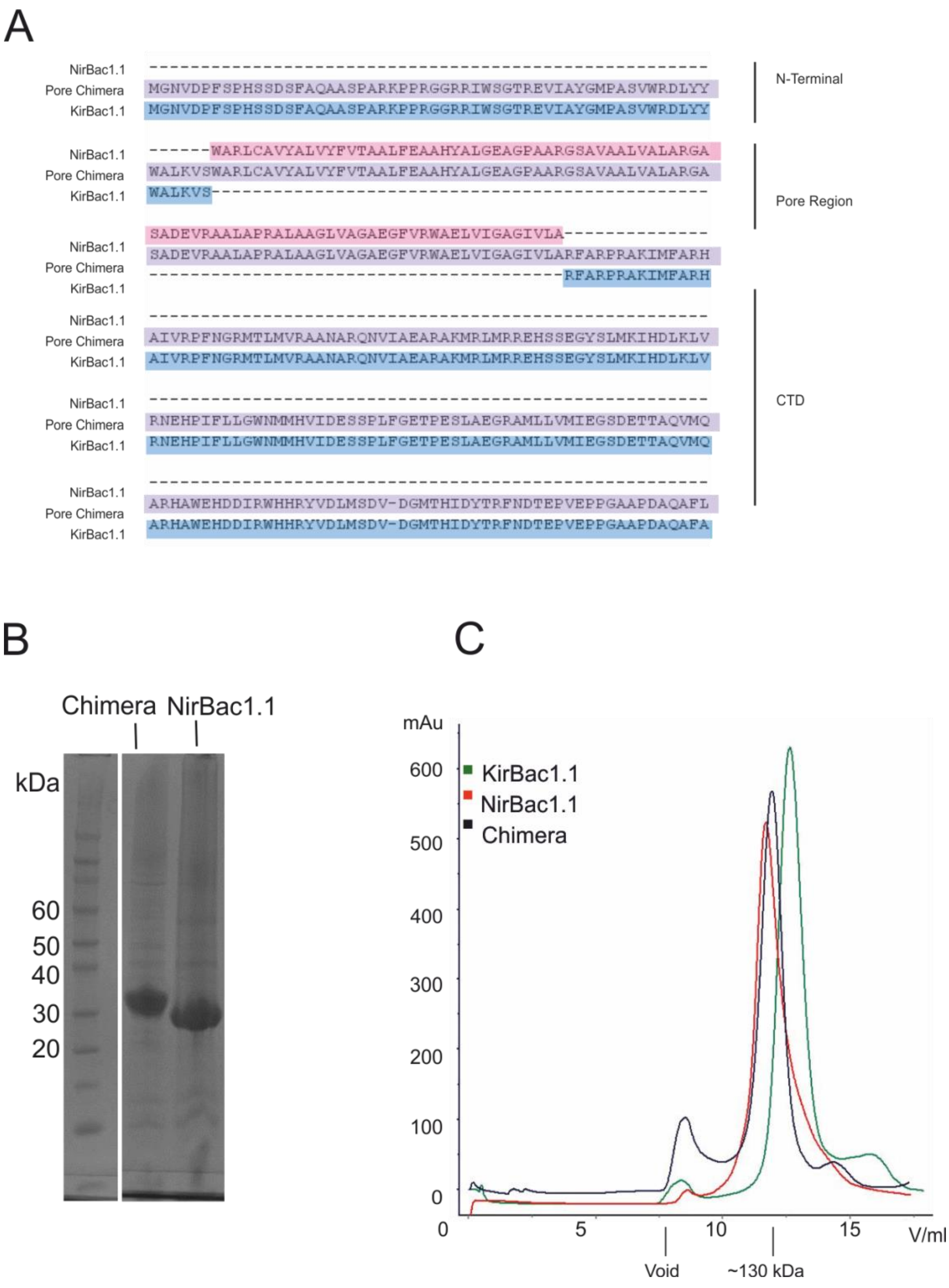

Figure S5 NirBac1.1/KirBac1.1 chimera. **A** The NirBac1.1/KirBac1.1 pore chimera was designed to contain the N- and C-terminal domains of KirBac1.1 and the TM domains, including the P-loop, of NirBac1.1. **B** The purified pore chimera migrates similarly to NirBac1.1 on SDS-PAGE. **C** The chimera elutes from the size exclusion column predominantly as a tetramer.
